# Cor triatriatum sinister with partial atrioventricular septal defect in a cat

**DOI:** 10.17221/91/2024-VETMED

**Published:** 2025-03-24

**Authors:** Woohyun Go, Wonkyu Park, Gunha Hwang, Soyon An, Hee Chun Lee, Tae Sung Hwang

**Affiliations:** Institute of Animal Medicine, College of Veterinary Medicine, Gyeongsang National University, Jinju, Republic of Korea

**Keywords:** congenital heart defect, echocardiography, feline

## Abstract

A 6-year-old female neutered Turkish Angora cat was referred due to tachypnoea. The patient was diagnosed with cardiomegaly at a local hospital during a health screening two years ago. Tachypnoea occurred one year ago. On physical examination, the patient presented with a respiratory rate of 72 breaths per minute and a systolic blood pressure of 70 mmHg. Thoracic radiographs revealed severe cardiomegaly, left atrium (LA) enlargement, right atrium (RA) enlargement, right ventricular enlargement, and dilation of pulmonary arteries and veins. An alveolar pattern was identified in the right and left cranial lung lobes. Echocardiography revealed a membrane that divided the LA into two chambers, a defect in the lower atrial septum, and elongation of anterior or posterior tricuspid valves (TV). However, septal TV was not observed. During systole, blood flow from LA to RA was confirmed through a defect in the atrial septum. During diastole, blood flow from LA to left ventricular was confirmed. These findings suggest cor triatriatum sinister (CTS) with partial atrioventricular septal defect (AVSD). This report describes echocardiographic diagnosis of CTS with partial AVSD in a cat.

Cor triatriatum sinister (CTS) is a rare congenital cardiac anomaly in cats. It is characterised by the division of the left atrium (LA) into two chambers due to an abnormal membrane, obstructing smooth blood flow from the LA to the left ventricle (LV) (Kienle 1998). The condition is referred to as CTS when it affects the LA in cats, while in dogs, it is generally termed cor triatriatum dexter (CTD) when the membrane is located in the right atrium (RA) (Kienle 1998). The abnormal membrane results from incomplete absorption of the common pulmonary vein into the LA, leading to the separation of the LA into proximal and distal chambers (Rodefeld et al. 1990; Kienle 1998; Krasemann et al. 2007). The proximal chamber is often referred to as the accessory LA chamber, whereas the distal chamber is the true LA chamber (Rodefeld et al. 1990).

CTS occurs in approximately 0.6% of cats (Tidholm et al. 2015). It may co-occur with other congenital cardiac anomalies such as atrial septal defect (ASD), pulmonary vein stenosis, aortic stenosis, persistent left cranial vena cava, tricuspid valve stenosis and atrioventricular septal defect (AVSD) (Koie et al. 2000; Choi and Hyun 2008). Left-sided heart failure can develop in CTS patients due to congestion in the LV (Koie et al. 2000). When concurrent defects such as ASD and AVSD are present, left-to-right shunting through the defect can lead to pulmonary hypertension, ultimately resulting in right-sided heart failure (Liu and Ettinger 1968; Nakao et al. 2011). Therefore, recognising concurrent conditions is critical. Echocardiography plays an essential role in diagnosing CTS. However, concurrent congenital heart diseases associated with CTS are rare. CTS combined with partial AVSD is particularly uncommon. Only one such case has been previously reported (Nakao et al. 2011).

This case report aims to describe the echocardiographic features of CTS with partial AVSD and evaluate the usefulness of echocardiography in diagnosing congenital heart disease.

## Case presentation

A 6-year-old, female neutered Turkish Angora cat was referred due to tachypnoea. The patient was diagnosed with cardiomegaly during a health screening at a local hospital two years ago. Tachypnoea was observed in the past year. It worsened recently. The sleeping respiratory rate of the cat was 40 breaths per minute.

**Figure 1:**
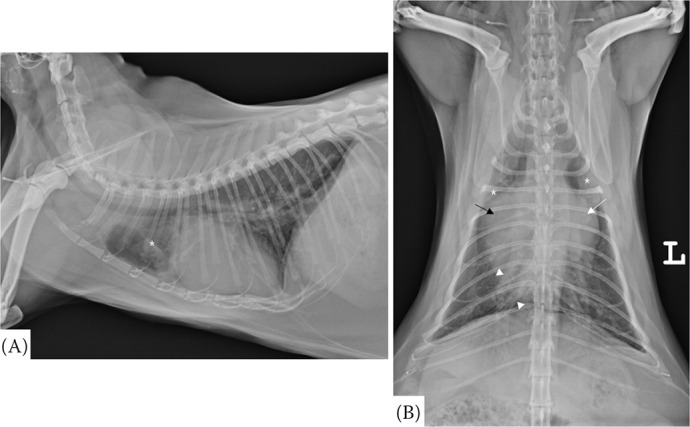
Right lateral (A) and ventrodorsal (B) thoracic radiographs showing severe cardiomegaly with enlargement of the left atrium (white arrow), right atrium (black arrow), right ventricle and dilation of the pulmonary arteries and veins (arrowheads) An alveolar pattern is observed in both the right and left cranial lung lobes (asterisks)

On physical examination, the cat exhibited a respiratory rate of 72 breaths per minute, a heart rate of 130 beats per minute, and a systolic blood pressure of 70 mmHg. Its complete blood count and serum chemistry profiles showed no significant abnormalities. Thoracic radiographs (Regius model 190; KONICA Minolta, Tokyo, Japan) showed severe cardiomegaly (vertebral heart score: 10; reference range: 6.7–8.1), bilateral atrial and right ventricular (RV) enlargement and dilation of pulmonary arteries and veins (Figure 1). An alveolar pattern was observed in the right and left cranial lung lobes, suggesting a congenital volume overload defect and pulmonary oedema.

**Figure 2:**
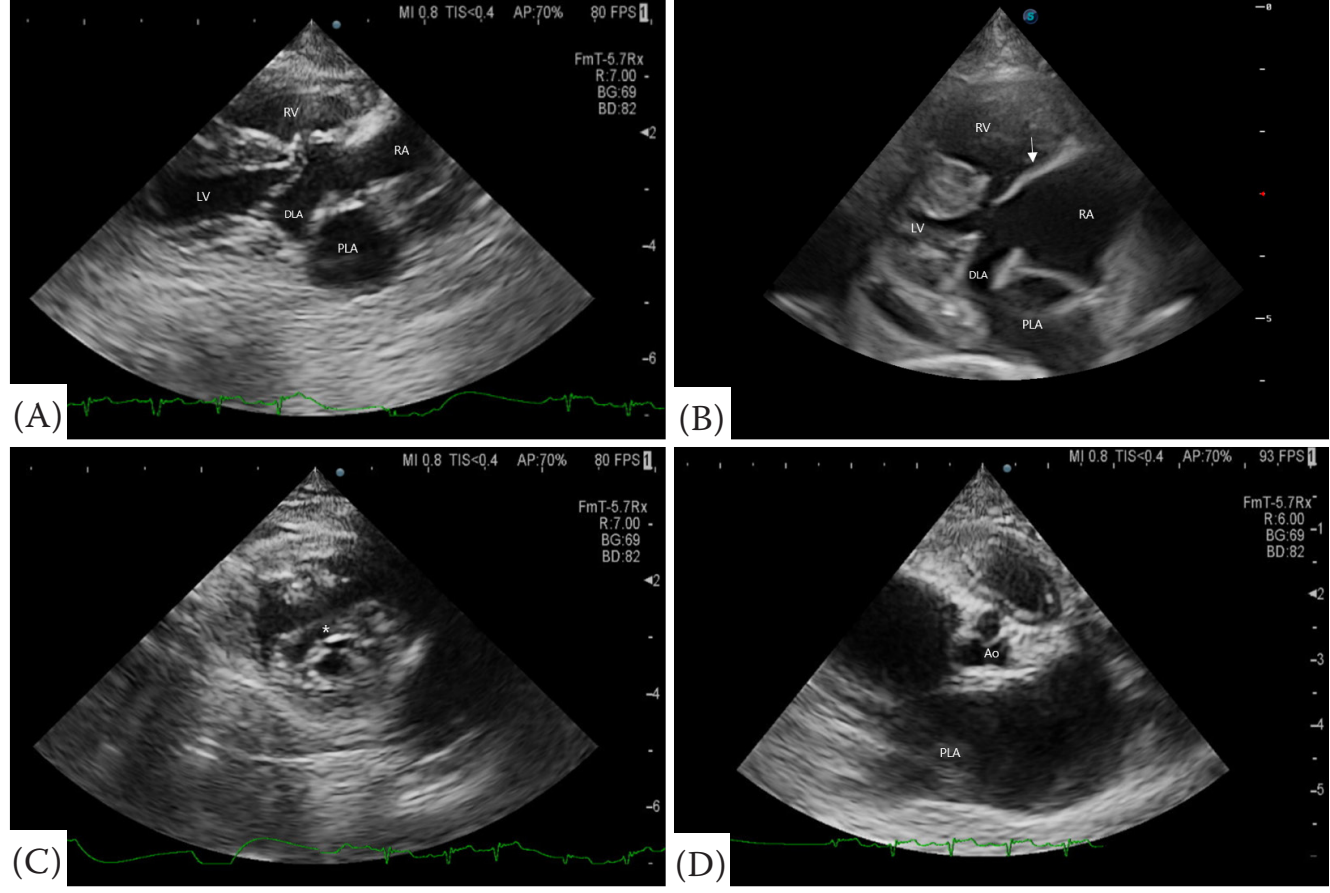
Two-dimensional echocardiography in the right parasternal 4-chamber (A, B) and short-axis views (C, D) (A) The echocardiographic view shows a membrane dividing the left atrium into two chambers, with the proximal chamber enlarged and the distal chamber reduced in size. (B) A large defect is visible in the lower part of the atrial septum, with elongation of the anterior or posterior tricuspid valve (TV) (arrow), although the septal TV is absent. (C) Flattening of the interventricular septum (asterisk). (D) Dilation of the proximal LA

**Figure 3:**
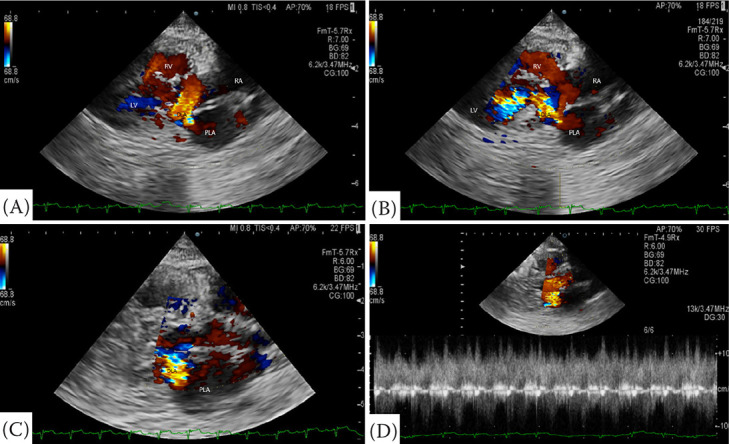
Colour-flow Doppler and continuous Doppler echocardiography images of the right parasternal 4-chamber view during systole and diastole (A) During systole, blood flow from the left atrium to the right atrium was confirmed through a defect in the atrial septum. (B) During diastole, blood flow from the left atrium to the left ventricle was observed. (C) Turbulent continuous blood flow was revealed in the distal left atrium chamber regardless of systole or diastole. (D) Turbulent continuous blood flow velocity was measured at 1.3 m/s with a pulsed-wave Doppler

**Figure 4:**
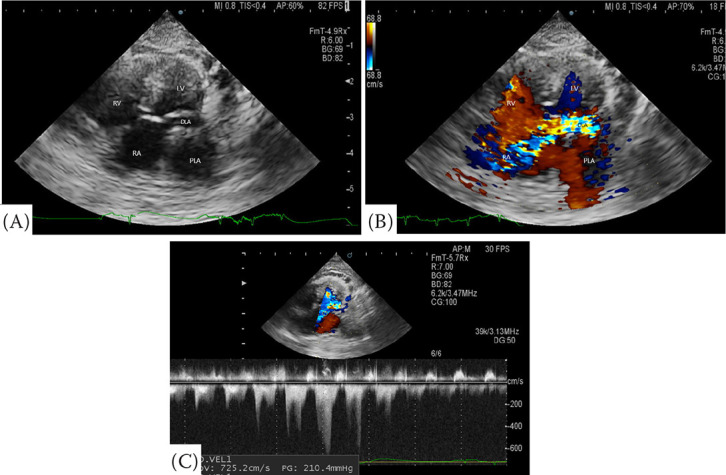
Two-dimensional (A), colour Doppler (B), and continuous wave Doppler (C) echocardiography images in a left-apical view During systole, the flow velocity from the left atrium to the right atrium was measured at 7.2 m/s in a left apical view

Echocardiographic examinations including transthoracic 2D, M-mode, spectral and colour-flow Doppler were conducted using a 2-9 MHz phased array transducer (Aloka Arietta 70; Hitachi Aloka, Wallingford, USA). Two-dimensional echocardiography confirmed the presence of a membrane dividing the LA into two chambers, with the proximal chamber enlarged and the distal chamber reduced in size (Figure 2A,B). A defect in the lower atrial septum and elongation of the anterior or posterior tricuspid valve (TV) were observed. However, the septal leaflet of the TV was absent (Figure 2B). Colour-flow Doppler revealed continuous turbulent blood flow in the distal LA chamber during both systole and diastole (Figure 3C,D). During diastole, blood flow from the LA to the LV was confirmed (Figure 3B). During systole, blood flowed from the LA to the RA via the atrial septal defect (Figure 3A). The interventricular septum appeared flattened, indicating increased pressure in the RV (Figure 2C). In the short-axis view, dilation of the proximal LA was evident (LA : Ao ratio = 2.29; proximal LA diameter = 17.7 mm). M-mode measurements yielded the following: left ventricular internal dimension at end-diastole (LVIDd) = 11.9 mm; left ventricular internal dimension at end-systole (LVIDs) = 8.3 mm; interventricular septum thickness at end-diastole (IVSd) = 5.0 mm; left ventricular posterior wall thickness at end-diastole (LVPWd) = 5.6 mm; fractional shortening = 29.8%; and ejection fraction = 61.7%. During systole, flow velocity from the distal LA to the RA was measured at 7.2 m/s in the left apical view (Figure 4B). Such high velocity could be attributed to the flow during systole where mitral regurgitation flowed from the distal LA into the RA. Additional measurements included E peak = 1.27 m/s; A peak = 0.52 m/s; and E: A ratio = 2.45. No regurgitation was detected in mitral, tricuspid, aortic, or pulmonic valves. Based on thoracic radiography and echocardiographic findings, a tentative diagnosis of CTS with partial AVSD was made.

The patient was treated with furosemide (1 mg/kg b.i.d.), benazepril (0.25 mg/kg b.i.d.) and clopidogrel (18.75 mg/cat s.i.d.) to manage congestive heart failure. After treatment, its respiratory rate was normalised. The cat remained in good condition for five months. However, six months later, it presented with lethargy and increased respiratory rate. Physical examination revealed a body temperature of 36.8 °C, a heart rate of 130 bpm, tachypnoea, and a blood pressure of 50–60 mmHg with mildly pale mucous membranes. Thoracic radiographs showed worsened cardiomegaly and pulmonary vessel dilation along with mild pleural effusion. At the owner’s request, euthanasia was performed.

## DISCUSSION

CTS is a rare congenital heart defect caused by an abnormal fibromuscular membrane that divides the LA into proximal and distal chambers (Kienle 1998). This membrane forms when absorption of the common pulmonary vein into the LA is incomplete during embryological development (Rodefeld et al. 1990; Kienle 1998; Krasemann et al. 2007). Clinical symptoms of CTS may include congestive heart failure, cyanosis, respiratory distress and fatigue. Physical examination findings include heart murmurs and a loud second heart sound (Rodefeld et al. 1990). In human medicine, CTS is classified into three types: Type A, where the proximal LA chamber receives all pulmonary veins and integrates with the LA; Type B, where the proximal LA chamber receives all pulmonary veins without integrating with the LA; and Type C, where the proximal LA chamber receives some pulmonary veins and integrates with either the LA or RA (Walsh et al. 2013). In our case, Type A CTS was considered as the accessory LA chamber was connected to the LA. However, additional vascular anomalies could not be confirmed due to the lack of CT angiography.

CTS may be associated with other congenital cardiac defects such as ASD, pulmonary vein stenosis, aortic stenosis, persistent left cranial vena cava, tricuspid valve stenosis and AVSD (Koie et al. 2000). CTS combined with AVSD is exceedingly rare (Nakao et al. 2011). In veterinary medicine, AVSD is classified into partial and complete forms (Santamarina et al. 2002; Yamano et al. 2011). Complete AVSD involves a large defect in both atrial and ventricular septa, along with a common atrioventricular valve. Partial AVSD presents with clefts or dysplasia in the anterior mitral valve or septal tricuspid valve along with an ostium primum ASD (Piccoli et al. 1979; Kienle 1998). Our case involved an ostium primum ASD and TV dysplasia without a ventricular septal defect, leading to a diagnosis of partial AVSD.

In cases of CTS, LA enlargement is typically observed on thoracic radiographs (Koie et al. 2000). However, in CTS with AVSD, both LA and right heart enlargement are commonly seen due to a left-to-right shunt (Nakao et al. 2011). In our case, both the LA and the right side of the heart were enlarged based on thoracic radiographs. The abnormal fibromuscular membrane in the LA obstructed smooth blood flow, leading to congestion and LA enlargement. During systole, blood flowed from the LA to the RA, causing congestion on the right side and contributing to right heart enlargement. This could explain the continuous turbulent flow observed during both systole and diastole in the distal LA chamber.

Previous reports on CTS with partial AVSD have suggested that left-to-right shunt caused by AVSD can increase pressure in the right heart, potentially leading to right-sided heart failure and death (Liu and Ettinger 1968; Nakao et al. 2011). In our patient, initial dilation of the proximal LA, pulmonary vessel enlargement and pulmonary oedema were signs of left-sided heart failure. The reduced blood flow into the LV during diastole and decreased systolic function likely impacted systemic circulation. A small amount of pleural effusion observed at the time of death indicated concurrent left heart failure.

In human patients, surgical treatment of CTS has been associated with a good prognosis, although other anomalies such as ASD may adversely affect outcomes (Oglietti et al. 1983; Rodefeld et al. 1990). Currently, treatment for CTS with AVSD in cats has not been established yet. However, treatment with furosemide and digoxin has been reported in the case of CTS with partial AVSD to alleviate cardiac congestion (Nakao et al. 2011). In our patient, diuretics were used to manage congestion.

Cats with CTS typically have a poor prognosis, often dying at an early age (Kienle 1998). However, the cat in our case lived until six years of age. In human cases with CTS and ASD, the ASD may act as a “pop-off” valve, relieving high pressure in pulmonary venous chambers caused by CTS (Lambert et al. 2017). In our case, the AVSD might have served a similar function. In conclusion, our patient lived longer than expected possibly due to the AVSD acting as a “pop-off” valve.

This case report provides detailed clinical and diagnostic imaging findings for CTS with partial AVSD in a cat. Echocardiography is proven to be a valuable tool for diagnosing and evaluating CTS with AVSD in patients suspected of having congenital heart defects.
